# Application of Machine Learning to Child Mode Choice with a Novel Technique to Optimize Hyperparameters

**DOI:** 10.3390/ijerph192416844

**Published:** 2022-12-15

**Authors:** Hamed Naseri, Edward Owen Douglas Waygood, Bobin Wang, Zachary Patterson

**Affiliations:** 1 Department of Civil, Geological, and Mining Engineering, Polytechnique Montréal, Montreal, QC H3T 1J4, Canada; 2 Department of Mechanical Engineering, Université Laval, Quebec, QC G1V 0A6, Canada; 3 Concordia Institute for Information Systems Engineering, Concordia University, Montreal, QC H3G 1M8, Canada

**Keywords:** children’s travel mode choice, multi-objective hyperparameter tuning, metaheuristic optimization, light gradient boosting machine

## Abstract

Travel mode choice (TMC) prediction is crucial for transportation planning. Most previous studies have focused on TMC in adults, whereas predicting TMC in children has received less attention. On the other hand, previous children’s TMC prediction studies have generally focused on home-to-school TMC. Hence, LIGHT GRADIENT BOOSTING MACHINE (LGBM), as a robust machine learning method, is applied to predict children’s TMC and detect its determinants since it can present the relative influence of variables on children’s TMC. Nonetheless, the use of machine learning introduces its own challenges. First, these methods and their performance are highly dependent on the choice of “hyperparameters”. To solve this issue, a novel technique, called multi-objective hyperparameter tuning (MOHPT), is proposed to select hyperparameters using a multi-objective metaheuristic optimization framework. The performance of the proposed technique is compared with conventional hyperparameters tuning methods, including random search, grid search, and “Hyperopt”. Second, machine learning methods are black-box tools and hard to interpret. To overcome this deficiency, the most influential parameters on children’s TMC are determined by LGBM, and logistic regression is employed to investigate how these parameters influence children’s TMC. The results suggest that MOHPT outperforms conventional methods in tuning hyperparameters on the basis of prediction accuracy and computational cost. Trip distance, “walkability” and “bikeability” of the origin location, age, and household income are principal determinants of child mode choice. Furthermore, older children, those who live in walkable and bikeable areas, those belonging low-income groups, and short-distance travelers are more likely to travel by sustainable transportation modes.

## 1. Introduction

Predicting travel mode choice is essential for transportation planning. However, most previous travel mode choice studies have focused on adults, whereas analyzing TMC in children has received less attention. Possibly, as a result, most transport planning is based on adults’ needs, and children are more and more reliant on adults for their transport needs [1]. Children are members of society, and travel mode choice has been found to relate to their overall wellbeing. Children travels can influence parents’ travel behavior [2]. Furthermore, children become adults, and their childhood behaviors can impact their adult behaviors. Therefore, it is important to examine children’s TMC and how different parameters impact healthier and more sustainable mode choices.

In real-life travel behavior, individuals choose between different transportation modes. Therefore, the features of different transportation modes (e.g., travel time, travel cost, and availability of different modes), as well as respondent characteristics, are used as factors influencing the choice between available alternatives [3]. Traditionally, different statistical methods, such as structural equation modeling [4], multivariate regression [5], bivariate analysis [6], and fuzzy logic [7], have been used to determine the factors influencing child travel behavior. For TMC, discrete choice models have been considered appropriate methods to analyze these kinds of datasets; hence, they have been widely employed [8,9,10].

Multinomial logit has been the most used discrete choice model to analyze travel behavior due to its simplicity and straightforward interpretation [11]. To this end, multinomial logit has been applied in many settings, such as daily TMC modeling [12], modeling the influence of new public transportation infrastructures on TMC [13], modeling shopping trips [8], and home-to-work/school commute modeling [9]. The use of the multinomial logit requires assuming error terms to be identically and independently distributed (IID), which can reduce prediction accuracy. Therefore, researchers have applied other discrete choice models to relax the IID assumption and obtain still more accurate results. In this regard, heteroscedastic extreme value, nested logit, mixed multinomial logit, multinominal probit [10,14,15], joint models, hybrid choice [16], and latent class discrete choice models [17] have been used to model individual travel mode choice.

Recently, due to the widespread application of machine learning techniques in various fields, these learning techniques have received growing attention for travel behavior modeling [18]. Machine learning techniques generally outperform discrete choice models when comparing the prediction accuracy [19,20,21,22]. Various machine learning techniques have been employed to predict TMC, such as support vector machines [23], random forest [24], naïve Bayes [25], extreme gradient boosting [26], kernel logistic regression [27], softmax regression [28], adaptive-neuro-fuzzy classification [29], k-nearest neighbor [30], and gradient boosting [20]. Although machine learning techniques have been found to be accurate techniques for prediction problems (e.g., TMC prediction), two challenges are associated with these techniques:

(a) Machine learning techniques require the determination of hyperparameters [31].
(b) Most of the powerful machine learning techniques are “black-box”, and, as a result, their results are not easily interpretable [32].

While ML techniques require the determination of hyperparameters, their determination is typically performed ad hoc. Table 1 provides a summary of recent studies on TMC prediction using machine learning techniques and the methods applied to tune hyperparameters. As can be seen, over 30% of those studies did not tune hyperparameters at all, and, as a result, their models might suffer from over- or underfitting. When hyperparameter tuning is undertaken, it is normally applied by breaking datasets into training, validation, and sometimes even testing datasets. Furthermore, the existence of excessive outliers on validation data can lead to selecting nonoptimal values for hyperparameters. To overcome these deficiencies, the application of k-fold cross-validation is recommended [31]. However, only 42.4% of studies shown in Table 1 employed the k-fold cross-validation process to tune hyperparameters.

The application of a robust method to tune hyperparameters is vital to develop an accurate prediction model. As shown in Table 1, trial and error is the most commonly used method in TMC prediction studies. However, the trial-and-error method has two major problems; it is a time-consuming technique and depends on modeler experience [53]. Accordingly, other researchers have applied systematic methods, including grid search, random search, and Hyperopt. Grid search is a brute force method, and it is not computationally efficient. Random search does not guarantee that optimal hyperparameters are found [54]. Moreover, all of these methods (i.e., trial and error, grid search, random search, and Hyperopt) only apply a single performance indicator (e.g., prediction accuracy) to tune hyperparameters. However, in many real-life prediction problems (e.g., TMC), datasets are not balanced. For instance, there tend to be many more car than bicycle trips (e.g., [52]). The promotion of such active modes may be a policy objective, but models using only overall accuracy might not adequately predict low-frequency modes. Thus, rather than simply using accuracy as a single performance indicator, multiple performance indicators (such as accuracy and F1-score together) should be applied to solve the problem of the imbalanced distribution of transportation modes. Hence, developing a new method that can consider multiple performance indicators in hyperparameter tuning is important, but currently overlooked.

As mentioned, the second problem with machine learning techniques is their black-box nature. To address this, many white-box prediction techniques have been developed, such as programming techniques (e.g., soccer league competition [55], water cycle programming [56], coyote optimization programming [32], and marine predator programming [57]) and M5tree [58]. Programming techniques cannot be applied to classification problems. Additionally, M5tree cannot represent the influence of variables on the response variable considering all respondents. In this regard, researchers have begun using ensemble machine learning techniques (e.g., gradient boosting) for TMC prediction problems since these methods can present the relative influence of each input variable on the response variable [20,24,26,52]. Although ensemble techniques can determine the influence of each variable, they can represent the direction of those influences.

Accordingly, after detecting the input variables with the highest relative influence on the response variable, different methods, such as accumulated local effects [59], Shapley additive explanations (SHAP) [60], partial dependence plot (PDP) [61], and local interpretable model agnostic explanations (LIME) [62], can be applied to represent in which the direction (positively, negatively, linearly, quadratically, etc.) of the top input variables impacts the response variable. However, LIME cannot indicate the influence direction of variables for all respondents, and it is a disaggregated technique. Although SHAP, PDP, and ALE can illustrate the influence direction of variables considering all data samples, they cannot represent whether the behavior of different groups is significantly different or not. Hence, multinomial logistic regression [63] is often used to determine the influence direction of variables and detect which groups significantly behave differently.

As can be seen from Table 1, although research has been conducted on adult TMC, child TMC has not received enough attention, and this group has been excluded in most studies. To address this issue, this study developed a model to predict the TMC of children and determine which variables significantly influence child mode choice for all trips (i.e., not only school-related trips) using an ensemble learning approach. Since conventional techniques to tune hyperparameters may not be highly efficient, and they can only optimize a single indicator during the tuning process, a new technique is proposed in this study that can optimize multiple indicators. The proposed technique can be highly effective for imbalanced datasets (e.g., Montreal TMC data), as the F1-score and accuracy can be maximized simultaneously. After detecting the most important variables on children’s TMC, multinomial logistic regression is applied to make the results of the black-box prediction technique interpretable. In other words, multinomial logistic regression is used to represent in which direction top-ranked variables can influence child TMC and how these variables can support sustainable transportation.

In the next section, the datasets used in this study is initially explained. Then, the developed technique and the conventional techniques applied for tuning hyperparameters are described. Afterward, the results are presented and discussed.

## 2. Methods

The main objectives of this studies are as follows:

- To develop a new method to tune hyperparameters;
- To predict child mode choices accurately;
- To determine which variables influence the child travel mode choice.

**Figure 1:**
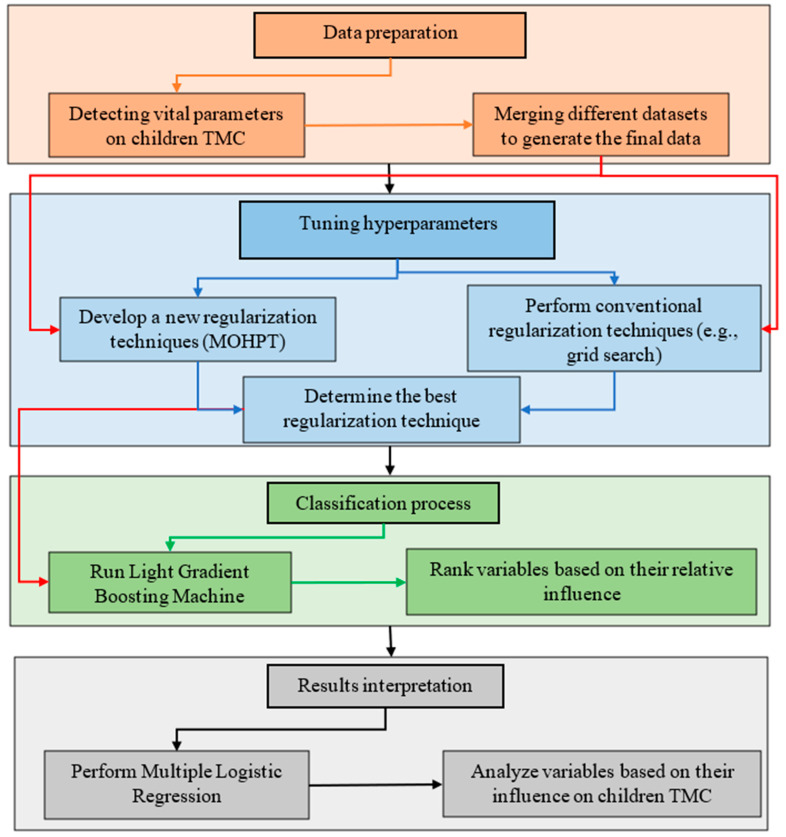
The flowchart of the methodology.

The flowchart of the methodology is shown in Figure 1. As can be seen, initially, different datasets are merged to develop a comprehensive dataset including many variables. Then, a new method is developed to tune hyperparameters. The developed method is compared with conventional regularisation techniques based on prediction accuracy and running time. The most accurate hyperparameter tuning technique is then used to run the final model. Subsequently, the machine learning technique is run, and the relative influence of variables is determined. Lastly, multiple logistic regression is used to interpret the results of the machine learning technique.

### 2.1. Datasets and Variables

To the best of the authors’ knowledge, most previous studies used trip details, individual and household characteristics to model TMC. However, in this study, additional variables, such as accessibility, geographic, and land-use variables, are added to the mentioned variables to help explain TMC. To this end, three datasets are taken into account, the 2018 Montreal OD survey, Walk Score, and Montreal proximity measure data.

The Montreal OD survey was conducted in the fall of 2018, and roughly 400,000 trips were recorded for “an average fall” day. From this survey, 14 variables are considered, including age, gender, availability of a monthly transit pass, disability status, interview language, household income, the presence of people in the household with restrictions in movement, number of members in the household, number of cars in the household, trip distance, start time of the trip, reason for trip, region of origin, and region of destination.

From the Walk Score dataset [64], walk score, transit score, and bike score variables were collected. Walk score measures the walkability of a location according to the distance to different amenities, including schools, parks, restaurants, grocery stores, and coffee shops. Transit score represents how well a location is served by public transit. The bike score indicates how a location is good for biking based on the availability of bike lanes, road connectivity, hilliness, and nearby amenities. These indices quantify the quality of walking, transit, and biking trips from 0 (worst) to 100 (excellent).

The built environment data were further enriched by using proximity data for Montreal. Ten variables were added, including accessibility level to primary school, secondary school, childcare facility, park, library, grocery store, health facility, pharmacy, employment source, and public transit. These indices measure the closeness of a dissemination block to the mentioned services using a gravity-based accessibility measure. Dissemination blocks are the smallest geographic area bounded on all sides by streets or boundaries of Statistics Canada’s standard geographic areas [65]. For more information about the mentioned accessibility indices, please visit Statistics Canada [66]. In contrast to walk score, which provides an overall score, these values are destination-specific.

Altogether, 27 variables were applied to explain TMC. The attributes of selected variables are shown in Table 2. Since this investigation focused on children’s TMC, the trips of individuals aged from 5 to 17 were taken into consideration (5 is the minimum age for trips to be collected on an individual level in Montreal; 18 is considered an adult in Canada). The trips where the origin is home were considered because the first trip’s mode restricts the following TMC [52], and built environment data were collected according to the individual’s residential location. In the final dataset, the number of relevant trips was 9597. These observations were randomly divided into training (80% of total samples) and testing data (20% of total samples). Six transportation modes were used for the mentioned trips: school bus (18.6%), car as a passenger (33.6%), bus (10.9%), rail transit (6.7%), cycling (2.4%), and walking (27.8%). The share of these modes in the dataset was 18.6%, 33.6%, 10.9%, 6.7%, 2.4%, and 27.8%, respectively. Hence, the share of transportation modes was imbalanced, and it was more appropriate to develop a model that can maximize F1-score, as well as accuracy, in the hyperparameter tuning process.

### 2.2. Modeling

For modeling TMC, an ensemble learning approach was applied for two reasons. First, the results of recent studies showed that ensemble prediction techniques generally outperform other modeling techniques, such as naïve Bayes, logistic regression, k-nearest neighbor, support vector machine, artificial neural network, nested logit, and multinomial logit, in explaining TMC in terms of prediction accuracy [11,30,50,52]. Second, ensemble techniques can prioritize variables on the basis of their relative influence on the response variable [67].

In this study, light gradient boosting machine (LGBM), a powerful and fast ensemble technique, was employed for the prediction process. LGBM is an updated version of tree-based gradient boosting developed by Microsoft. Like other ensemble techniques, LGBM combines different weak learners (i.e., decision trees) to form a powerful and robust prediction algorithm [68]. LGBM is a quick method, and it is highly efficient for large-scale prediction problems. Parallel learning is supported by LGBM, and, as a result, memory usage is significantly reduced. A leaf-wise leaf growth strategy is used in LGBM modeling that can limit the depth growth in the splitting process. The mentioned leaf-wise leaf growth strategy splits the same layer of leaves simultaneously. Therefore, LGBM can implement multithreaded optimization. To this end, the complexity of the model is controlled automatically, and the probability of overfitting is considerably reduced [69].

### 2.3. Tuning Hyperparameters

A new technique is proposed to optimize hyperparameters of machine learning techniques considering multiple performance indicators. That is, a new multi-objective hyperparameter tuning (MOHT) approach is developed in this study. In this regard, non-dominated sorting genetic algorithm III (NSGA-III), a multi-objective metaheuristic algorithm, was used as the optimization tool. Genetic algorithms have been widely used to optimize several engineering problems [70,71,72]. NSGA-III was used for the optimization process since it is a multi-objective metaheuristic optimization technique, and metaheuristic techniques can sync with machine learning techniques [73]. In this technique, the hyperparameter values are optimized using an optimization framework. In each iteration of the optimization process, NSGA-III assigns different values to hyperparameters. Then, the machine learning technique is run to evaluate the performance indicators (i.e., accuracy and F1-score) for each of the assigned hyperparameters. Then, the model tries to improve the performance indicators by optimizing the hyperparameters. The optimization modeling of the proposed method is presented in Equations (1)–(5).

(1) $$Z_{1}{=\mathrm{maximize}\;\mathrm{Accuracy}}^{K-CV},$$

(2) $$Z_{2}=\mathrm{maximize}\;F_{1}^{K-CV},$$

(3) $$HP_{i}^{Int}\in Set_{i}\;\;\forall i\in I,$$

(4) $$HP_{j}^{Con}\geq HP_{j}^{\min}\;\;\forall j\in J,$$

(5) $$HP_{j}^{Con}\leq HP_{j}^{\max}\;\;\forall j\in J,$$

where $Z_{1}$ and $Z_{2}$ are the objective functions of the proposed optimization model. ${\mathrm{Accuracy}}^{K-CV}$ and $F_{1}^{K-CV}$ imply the accuracy and F1-score of validation data calculated using the k-fold cross-validation technique. In this study, a fivefold cross-validation was used for the tuning process (*K* = 5). $HP_{i}^{Int}$ and $HP_{j}^{Con}$ denote integer and continuous-ranged hyperparameters. $Set_{i}$ is the defined set of integer hyperparameter i. $HP_{j}^{\min}$ and $HP_{j}^{\max}$ are the minimum and maximum defined values for continuous-ranged hyperparameters j. I and J represent the number of integer and continuous-ranged hyperparameters, respectively.

Equations (1) and (2) are the objective function of the proposed technique. That is, in the hyperparameter tuning process, accuracy and F1-score are maximized simultaneously. Equation (3) guarantees that the optimal value of integer hyperparameters is selected from their defined set. Equations (4) and (5) are the constraints that force the model to select the optimal value of continuous-ranged hyperparameters from their allowed range.

**Figure 2:**
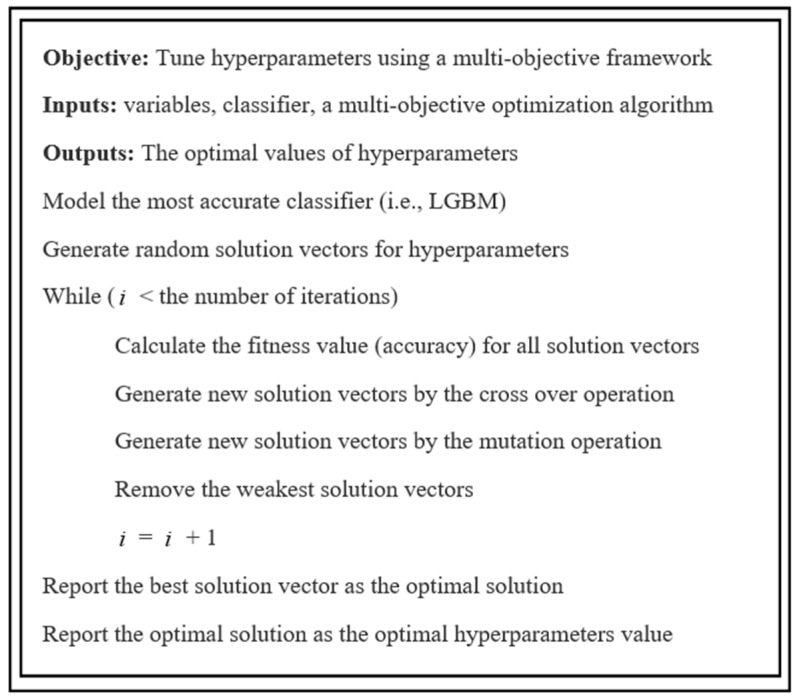
The pseudo-code of MOHT.

As mentioned, NSGA-III was employed to solve the multi-objective optimization problem. NSGA-III is a metaheuristic optimization algorithm that is used for solving multi-objective optimization problems. This algorithm aims to find non-dominated sorting optimal solutions integrating all objective functions rather than converting all objective functions into a single objective function. As a result, NSGA-III presents a Pareto front in which the optimal solutions cannot dominate each other on the basis of all objective functions [74]. The pseudo-code of MOHT is shown in Figure 2.

Three conventional hyperparameter tuning techniques, namely, grid search, random search, and Hyperopt, were used to evaluate the effectiveness of the proposed hyperparameter tuning approach (i.e., MOHT). Grid search checks all the possible combinations of hyperparameters to find their optimal values. That is, a possible set for each hyperparameter should be defined. Then, all the possible combinations of hyperparameters in the possible set are used to run the model. Lastly, the combination that leads to the highest accuracy is considered the optimal value of hyperparameters. Random search only checks some random possible combinations of hyperparameters and tunes hyperparameters on the basis of a limited number of random combinations. Hyperopt is an efficient hyperparameter tuning method that applies parallel and serial optimization to efficiently optimize hyperparameters [75].

Grid search is a brute force technique, and it only assigns a limited number of possible hyperparameter values to the hyperparameters’ initial set. That is, for hyperparameters with a continuous range, only a few values can be checked, and the optimal value of hyperparameters may not be found. However, grid search is an exact algorithm, and its optimal solution is not changed in different runs. Random search may not find the optimal values for hyperparameters because it assigns random values to hyperparameters. Nonetheless, random search is a quick technique, and it is computationally efficient when the number of hyperparameters is significant. Hyperopt is computationally more efficient than grid search, while its running time is generally higher than random search. Furthermore, all these techniques apply a single performance indicator (e.g., accuracy) to tune hyperparameters. To address this issue, this study developed the MOHT.

The defined set for hyperparameters is presented in Table 3. As can be seen, grid search cannot cover the entire range, and a set with a few possible options should be considered in this technique since it is a comprehensive search method.

### 2.4. Results Interpretation

Although LGBM can rank variables on the basis of their relative influence on the response variable (i.e., children’s TMC), it cannot interpret how each variable (e.g., trip distance) impacts the children’s TMC. To solve this issue, after detecting the variables with the highest relative influence on children’s TMC, multinomial logistic regression was applied to determine how these top-ranked variables influence the children’s TMC. Since multinomial logistic regression cannot converge when the number of variables is significant, the top variables on child TMC were detected using the relative influence presented by LGBM. Then, those top variables were applied for modeling multinomial logistic regression.

Multinomial logistic regression is a robust statistical modeling technique that can be used for classification and interpretation. A set of explanatory variables are used in multinomial logistic regression for assessing the probability of dichotomous outcome events. Dichotomous variables mainly represent whether some events occur or not. In this technique, it is assumed that the relation between the explanatory variables is linear. Therefore, multinomial logistic regression uses linear decision boundaries, but it is a nonlinear technique [67]. From a sustainable transport perspective, the car as a passenger is considered the reference in the multinomial logistic regression to determine how top variables can attract children to use more sustainable transportation modes.

## 3. Results and Discussion

In this section, the results of hyperparameter tuning techniques are initially presented, and the best technique is determined. Then, the ranking of variables based on their relative influence on children’ TMC is presented using the most accurate hyperparameter technique and LGBM. Ultimately, the results of a multinomial logistic regression model are presented.

### 3.1. The Performance of Hyperparameter Tuning Techniques

The optimal values of hyperparameters using different techniques are shown in Table 4. Although MOHT is a multi-objective algorithm and generally provides the users with multiple non-dominated optimal solutions (i.e., a Pareto front), it presents a single optimal solution for the applied case study. If MOHT presents over one optimal solution, it is recommended to apply gray relational analysis to find the best optimal solution according to the details provided by Naseri et al. [76].

**Figure 3:**
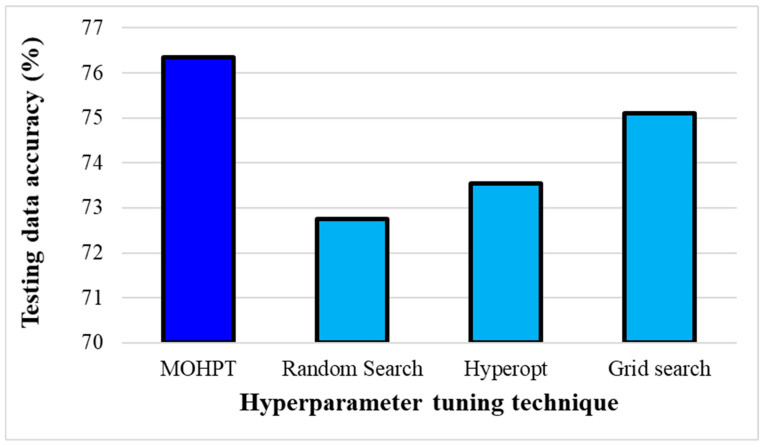
The testing data accuracy of hyperparameter tuning techniques.

**Figure 4:**
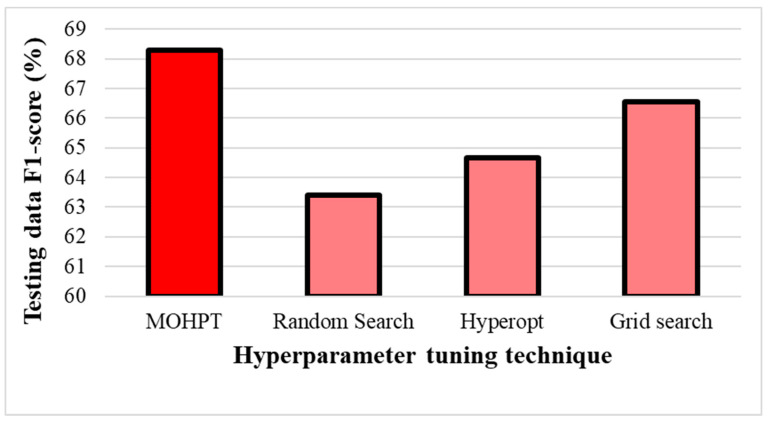
The testing data F1-score of hyperparameter tuning techniques.

The testing data accuracy and testing data F1-score of different hyperparameter tuning techniques are shown in Figure 3 and Figure 4. As can be seen, the proposed technique in this study (MOHT) obtained the highest testing data accuracy, followed by grid search, Hyperopt, and random search. That is, applying MOHT could increase the prediction accuracy by 1.25%, 2.81%, and 3.59%, respectively, compared to grid search, Hyperopt, and random search. Similarly, MOHT outperformed other techniques in terms of testing data F1-score. The testing data F1-score of MOHT was 1.74%, 3.61%, and 4.89% greater than that of grid search, Hyperopt, and random search, respectively. Therefore, the testing data F1-score improvement of MOHT was more than its prediction accuracy, which is related to considering both accuracy and F1-score in the objective function of MOHT. Hence, it can be postulated that considering multiple performance indicators in the tuning hyperparameter techniques can improve the overall performance of the model. However, techniques including a single performance indicator can only improve the prediction accuracy and not all vital performance indicators.

**Figure 5:**
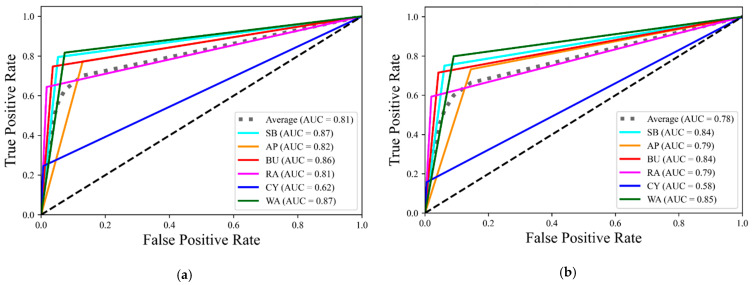

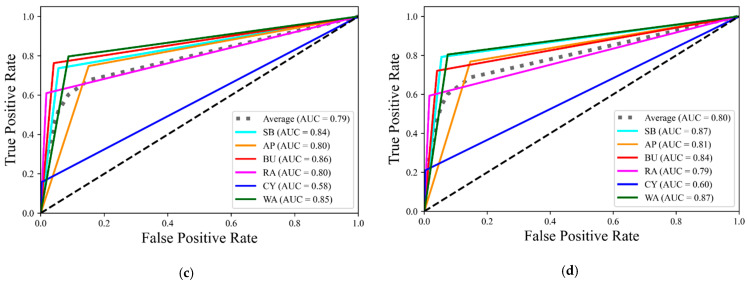
The area under the curve of receiver operating characteristic curves: (**a**) MOHT, (**b**) random search, (**c**) Hyperopt, and (**d**) grid search; SB: school bus, AP: automobile as a passenger, BU: bus, RA: rail transit, CY: cycling, WA: walking.

The receiver operating characteristic (ROC) curves of different hyperparameter tuning techniques are indicated in Figure 5. Drawing on the results, the highest area under the curve (AUC) of the ROC curves was related to MOHT, followed by grid search, Hyperopt, and random search, with values of 0.81, 0.80, 0.79, and 0.78, respectively. Accordingly, MOHT was the best technique. MOHT obtained the highest AUC of the ROC curve for the least frequent mode (cycling) with a value of 0.62, which was 2%, 4%, and 6% more than that of grid search, Hyperopt, and random search. This improvement resulted from considering prediction accuracy and F1-score in MOHT, proving that MOHT was highly efficient for modeling this imbalanced TMC dataset. Therefore, considering an optimization framework to tune hyperparameters can even improve the performance indicators not considered in the objective function of the optimization model.

**Figure 6:**
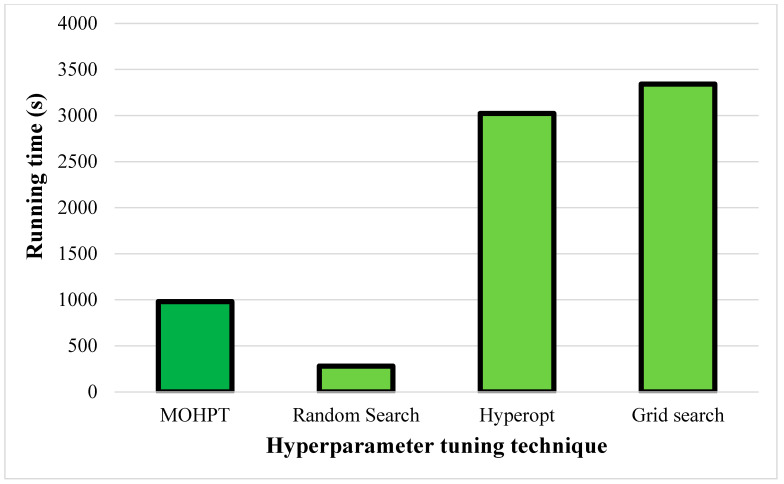
The running time of hyperparameter tuning techniques.

The running time of the hyperparameter tuning techniques is presented in Figure 6. MOHT could reduce the computational time by 68% and 71% compared to Hyperopt and grid search, indicating that MOHT was a highly efficient technique regarding the computational cost. However, the MOHT running time was 2.5 times more than that of the random search. As mentioned, random search checks a limited number of random combinations; hence, it was the fastest technique. On the other hand, random search is less likely to find optimal values of hyperparameters, and the testing data accuracy and F1-score obtained by the random search were significantly lower compared to MOHT. Therefore, it can be postulated that MOHT outperformed other techniques when considering the testing data accuracy, testing data F1-score, and running time.

Liashchynskyi and Liashchynskyi [54] compared the performance of grid search and random search regarding the prediction accuracy and running time. The results suggested that, although random search was a faster technique, grid search could obtain higher prediction accuracy. Hence, their results are in line with the findings of this study.

### 3.2. The Relative Influence of Variables on Children’s TMC

**Figure 7:**
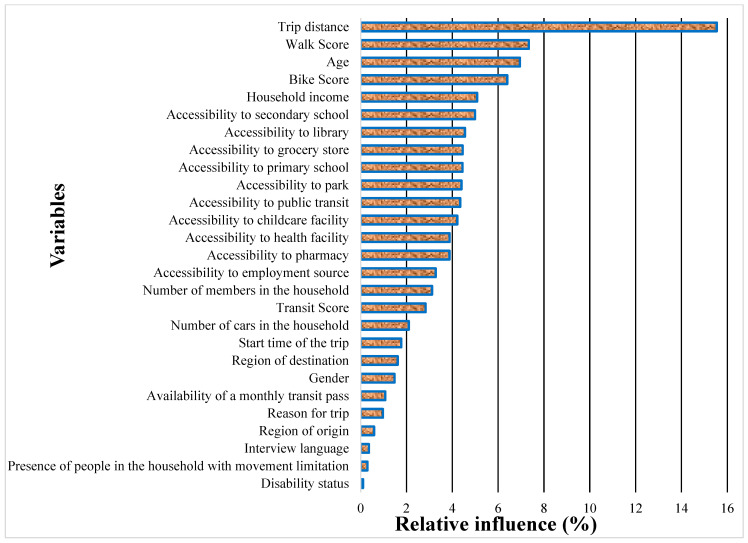
The relative influence of variables on children’s TMC.

Since MOHT led to the highest prediction accuracy, the LGBM was performed using the optimal hyperparameter values found by MOHT. Then, the relative influence of variables was extracted to determine which variables impact the children’s TMC the most. The relative influence is illustrated in Figure 7. As can be seen, trip distance had by far the highest impact on children’s TMC, with a relative influence of 15.5%. Walk score, age, bike score, household income, and accessibility to secondary school were the next best variables. The relative influence of other variables was less than 5%. Among accessibility parameters, accessibility to secondary school, accessibility to libraries, and accessibility to grocery stores had the greatest influence on children’s TMC.

Wang and Ross [77] investigated the relative influence of different variables on adults’ TMC, and the results suggested that the relative influence of trip distance was significantly higher than the number of vehicles per capita, population density, and the number of people in the household. Accordingly, their results are in line with the results of the current study. In the Kim [11] study, age had a considerably higher influence than gender in terms of relative influence on TMC, which is consistent with the results shown in Table 5.

### 3.3. Analyzing the Influence Direction of Top Variables

Although LGBM can rank the variables on the basis of their relative influence on the response variable, it cannot determine how changing variables affect the response variable. In this regard, multinomial logistic regression was performed to examine the direction influence of top-ranked variables, and the results are shown in Table 5. In the mentioned analysis, the car as a passenger was considered the reference. According to the results, most of the variables were statistically significant in terms of impact on children’s TMC, which may be related to considering the top-ranked variables of LGBM in the multinomial logistic regression.

Children are more likely in Montreal to travel by public transit (i.e., rail transit and bus) than by car as a passenger. Nonetheless, they are less likely to travel by school bus or active transportation (i.e., cycling and walking) then by car as a passenger. By reducing the trip distance, children are more likely to walk or cycle to their destination, while the probability of traveling by public transit or school bus is reduced. In regions with a lower walk score, children are more likely to travel by school bus, and public and active transportation are less used than cars as a passenger. Children aged under 12 years are more likely to travel by car as a passenger. For those aged over 15, rail transit was their preference, followed by cycling, bus, and walking. However, they were not likely to prefer the school bus over a car as a passenger. A reduction in bike score led to a reduction in the probability of choosing bus, rail transit, cycling, and walking over a car as a passenger. Furthermore, a statistically significant difference was not found between the car as a passenger and the school bus if bike score was changed.

As compared to high-income households, children in the low-income group (<60 thousand CAD annually) preferred the school bus, bus, rail transit, cycling, and walking over a car as a passenger. The middle-income group (60–120 thousand CAD annually) was more likely to travel by school bus as compared to the high-income group, but a significant difference could not be seen between a car as a passenger and other transportation modes. A reduction in the accessibility to secondary school resulted in an increment in the intention to choose school bus, bus, and walking over a car as a passenger. On the other hand, reducing the accessibility to secondary school decreased the probability of choosing rail transit and cycling over a car as a passenger.

### 3.4. Managerial Implications

Individuals over 15, those who live in regions with higher walk score, bike score, and accessibility to secondary schools, the low-income group, and short-distance travelers are more likely to travel by active transportation. Moreover, older children (aged over 15), long-distance travelers, residents of regions with higher walk score and bike score, and the low-income group generally use public transit more than a car as a passenger. children aged 12 to 15, residents of regions with the lowest level of walk score, bike score, and accessibility to secondary schools, long-distance travelers, and low- and middle-income groups are more likely to travel by school bus than by car as a passenger.

Therefore, improving the walk score can increase the share of active and public transportation in child trips. Similarly, the bike score needs to be increased if the goal is to promote active transportation in children. Accessibility to schools should be improved if the governments tend to attract children to travel by active transport.

One of the limitations of this study is that it only applied the NSGA-III algorithm to develop a multi-objective hyperparameter tuning technique. It is recommended to consider other multi-objective optimization algorithms to develop new hyperparameter tuning techniques and compare their accuracy with the method proposed in this study.

## 4. Conclusions

In this study, the travel mode choice of children aged 5 to 17 was investigated using a robust ensemble learning technique, LGBM. To maximize the model’s performance, a new multi-objective approach (MOHT) was proposed to tune machine learning techniques hyperparameters. The performance of the proposed technique was compared with the conventional tuning methods. MOHT was demonstrated to be an appropriate technique for tuning hyperparameters of imbalanced datasets (such as travel mode choice) since it can consider multiple machine learning performance indicators in the tuning process. MOHT outperformed other hyperparameter tuning techniques in terms of machine learning performance indicators (e.g., prediction accuracy, F1-score, and AUC). Moreover, this technique could significantly reduce the computational cost compared to grid search and Hyperopt. However, the running time of MOHT was considerably higher than the random search, but it could present more accurate solutions.

The independent variables were ranked on the basis of their relative influence on children’s TMC, and trip distance, walk score, age, bike score, household income, and accessibility to secondary schools were the top-ranked variables. Since LGBM could not represent how these top-ranked variables influence children’s TMC, multiple logistic regression was applied to better understand the influence of these variables on children’s TMC. With reference to trips by car, the results suggested that, as trip distance decreases, active modes are more likely. The built environment, as measured by walk score, was positively associated with all sustainable and independent modes as was bike score to a lesser degree. As age increased, children used more sustainable and independent modes. Finally, the highest household income was associated with more car as passenger trips, but the relationship with active modes was less strong. The results suggest that policies for a mixed-use development with high-quality public transport networks, such as Singapore’s 20 min towns and 45 min city [78], can facilitate both local travel and the use of public transport by children.

## Figures and Tables

**Table 1:** A summary of recent studies on the application of machine learning to travel mode choice prediction.

Reference	Optimizing Hyperparameters	Considering k-Fold Cross-Validation	Method for Tunning Hyperparameters	Children Mode Choice
Pham et al. [21]	✓	✓ (Tenfold)	Trial and error	✕
Pineda-Jaramillo and Arbeláez-Arenas [20]	✓	✓	Random search	✕
Kashifi et al. [33]	✕	✓ (Tenfold)	-	✕
Salas et al. [22]	✓	✓ (Fivefold)	Hyperopt	✓
Chao [25]	✕	✕	-	✕
Brenner et al. [34]	✕	✓ (Fivefold)	-	✕
Mohd Ali et al. [35]	✓	✕	-	✕
Li et al. [36]	✕	✕	-	✕
Tariq and Shakeel [37]	✓	✕	Trial and error	✓
Aschwanden et al. [38]	✕	✕	-	✕
Buijs et al. [39]	✓	✕	Random search	✕
Liu et al. [26]	✓	✓ (Fivefold)	Grid search	✕
Lu et al. [40]	✓	✕	Trial and error	✕
Sun and Wandelt [41]	✓	✕	Trial and error	✕
Kim [11]	✓	✕	Grid search	✕
Martín-Baos et al. [27]	✓	✓ (Twofold)	Random search	✕
Gao et al. [42]	✓	✓ (Tenfold)	Trial and error	✕
Qian et al. [43]	✓	✓ (Tenfold)	?	✕
Mi et al. [28]	✓	✓ (Fivefold)	Grid search	✕
Liang et al. [44]	✕	✕	-	✕
Mohd Ali et al. [30]	✕	✕	-	✕
Nam and Cho [45]	✓	✕	Trial and error	✕
Thanh et al. [46]	✕	✕	-	✕
Zhou et al. [47]	✓	✓ (Threefold)	Grid search	✕
Chang et al. [24]	✓	✓ (Fivefold)	Grid search	✓
Yang and Ma [48]	✕	✕	-	✕
Pirra and Diana [49]	✓	✓ (Fivefold)	?	✕
Richards and Zill [50]	✓	✓ (Tenfold)	Grid search	✓
Cheng et al. [51]	✓	✕	Trial and error	✕
Chapleau et al. [52]	✓	✕	Trial and error	✓
Minal et al. [29]	✓	✕	Trial and error	✕
Assi et al. [23]	✓	✕	Trial and error	✓

✓: yes; ✕: no; ?: could not find the in the manuscript.

**Table 2:** Attribute of selected variables.

Variable	Frequency	Percentage	Variable	Frequency	Percentage
Gender			Household income (CAD)		
Male	4989	51.98	Less than 30,000	543	5.66
Female	4608	48.02	30,000 to 59,999	1189	12.39
Availability of a monthly transit pass			60,000 to 89,999	1654	17.23
Yes	2118	22.07	90,000 to 119,999	1856	19.34
No	7479	77.93	120,000 to 149,999	1042	10.86
Disability status			150,000 to 179,999	603	6.28
Yes	84	0.88	180,000 to 209,999	376	3.92
No	9513	99.12	210,000 and more	713	7.43
Number of cars in the household			Refusal	1185	12.35
1	3669	38.23	Unknown	436	4.54
2	4532	47.22	Number of members in the household		
3	686	7.15	1	4	0.04
4	129	1.34	2	299	3.12
5	31	0.32	3	1484	15.46
6	5	0.05	4	4543	47.34
7	0	0.00	5	2412	25.13
8	1	0.01	6	604	6.29
9	0	0.00	7	170	1.77
10	0	0.00	8	50	0.52
11	0	0.00	9	16	0.17
12	0	0.00	10 and more	15	0.16
13	3	0.03	Interview language		
14 and more	0	0.00	French	8988	93.65
			Other	609	6.35

**Table 3:** Set of hyperparameters for various hyperparameter tuning techniques.

	MOHT	Random Search	Hyperopt	Grid Search
Number of estimators	{10, 11, 12, …, 510}	{10, 11, 12, …, 510}	{10, 11, 12, …, 510}	{10, 50, 100, 200, 300, 400, 500, 600}
Maximum depth	{1, 2, 3, …, 11}	{1, 2, 3, …, 11}	{1, 2, 3,…, 11}	{1, 3, 5, 7, 9, 11}
Minimum data in leaf	{5, 6, 7, …, 105}	{5, 6, 7, …, 105}	{5, 6, 7, …, 105}	{10, 20, 30, 40, 50, 60, 70}
Learning rate	(0, 1]	(0, 1]	(0, 1]	{0.0001, 0.001, 0.01, 0.1, 1}

**Table 4:** The optimal values of hyperparameters.

	Number of Estimators	Maximum Depth	Minimum Data in Leaf	Learning Rate
Definition of Hyperparameters	The Number of Decision Trees	The Maximum Depth of the Tree	The Minimum Data Required to Be at a Leaf Node	Convergence Magnitiude
MOHT	172	10	29	0.319
Random search	50	9	60	0.481
Hyperopt	429	10	54	0.028
Grid search	500	11	10	0.1

**Table 5:** Results of multiple logistic regression (variables are organized by relative influence from most to least).

		School Bus		Bus		Rail Transit		Cycling		Walking	
		Estimate	SL	Estimate	SL	Estimate	SL	Estimate	SL	Estimate	SL
Constant		−1.121	***	0.489	***	1.509	***	−1.899	***	−2.269	***
Trip distance (km)	0–0.8	−0.918	***	−1.693	***	−4.928	***	2.246	***	5.214	***
	0.8–1.6	−0.195	*	−0.585	***	−2.610	***	1.942	***	3.548	***
	1.6–3.2	0.042		0.033		−1.309	***	1.187	***	1.549	***
	>3.2 (ref)	0		0		0		0		0	
Walk Score	0–46	1.031	***	−1.240	***	−1.768	***	−0.028		−0.773	***
	46–70	0.580	***	−0.685	***	−1.341	***	−0.731	**	−0.603	***
	70–85	0.184	+	−0.247	*	−0.716	***	−0.693	***	−0.412	***
	86–100	0		0		0		0		0	
Age	5–8	−0.800	***	−3.432	***	−3.358	***	−2.147	***	−1.683	***
	9–11	−0.482	***	−2.621	***	−2.107	***	−1.359	***	−1.008	***
	12–15 (ref.)	0		0		0		0		0	
	16–17	−0.503	***	0.570	***	0.913	***	0.662	***	0.415	**
Bike Score	0–59	0.115	+	−0.625	***	−1.310	***	−1.503	***	−0.718	***
	60–71	−0.100	+	−0.349	*	−1.137	***	−1.321	***	−0.726	***
	72–84	0.101	+	−0.179	+	−0.870	***	−0.631	**	−0.614	***
	85–100 (ref)	0		0		0		0		0	
Household income (thousand CAD)	<60	0.771	***	0.623	***	0.487	**	0.170	+	0.073	+
	60–120	0.315	***	0.032		0.129	+	−0.030		−0.003	
	>120 (ref)	0		0		0		0		0	
	Prefer not to answer	0.432	***	0.140	+	0.506	***	−0.224	+	−0.102	+
Accessibility to secondary school	<0.048	0.286	**	0.144	+	−0.685	***	−0.255	+	0.103	+
	0.048–0.072	0.222	*	0.140	+	−0.919	***	−0.577	**	0.002	
	0.072–0.114	0.170	+	0.091	+	−0.307	*	−0.160	+	0.136	+
	>0.114 (ref)	0		0		0		0		0	

Nore: SL= significance level; +, *, **, and *** imply a significance difference at the levels of 0.1, 0.05, 0.01, and 0.001, respectively.

## Data Availability

Due to privacy issues, the data may not be shared publicly.
